# After forty-eight years: An enigmatic new wormlion fly from Xizang, China (Diptera, Vermileonidae)

**DOI:** 10.3897/zookeys.1276.184675

**Published:** 2026-04-07

**Authors:** Li-Xia Shan, Ji-Shen Wang

**Affiliations:** 1 College of Agriculture and Biological Science, Dali University, Dali, Yunnan 671003, China Cangshan Forest Ecosystem Observation and Research Station of Yunnan Province, Dali University Dali China; 2 Cangshan Forest Ecosystem Observation and Research Station of Yunnan Province, Dali University, Dali, Yunnan 671003, China College of Agriculture and Biological Science, Dali University Dali China

**Keywords:** Dinggyê, new species, taxonomy, western China, Yadong

## Abstract

The brachyceran family Vermileonidae (wormlion flies) is characterised by larvae that construct pitfall traps for predation. The Oriental genus *Vermitigris* Wheeler, 1930 previously included four described species distributed in China, India, Indonesia, and Malaysia. In 1978, unidentified larvae of *Vermitigris* were collected by Fa-Sheng Li from Yadong, Xizang, China, but the adult stage remained unknown. During a 2025 expedition, adult specimens were obtained, enabling their association with the larvae and recognition as a new species. Herein, *Vermitigris tsangyanggyatso***sp. nov**. is described, with accounts of its immature stages and notes on its biology. This discovery increases the number of *Vermitigris* species recorded from China from one to two, the total number of species in the genus from four to five, and all the extant species in the family from 66 to 67. The biogeographical implications for *Vermitigris* are also discussed.

## Introduction

The brachyceran family Vermileonidae previously comprised 13 genera, including two extinct monotypic genera (†*Protovermileo* Hennig, 1967 and †*Crevermileo* Feng, Ren & Wang, 2025), and 66 extant species in 11 genera worldwide (Bueno et al. 2021; Kehlmaier 2021; Li et al. 2024; Feng et al. 2025; Sun et al. 2026). Members of the family are widely distributed across Africa, Eurasia, and North America (Santos 2008; Bueno et al. 2021).

Vermileonid larvae, commonly known as wormlions, employ a pitfall-feeding strategy convergent with that of pitfall-building antlions (Neuroptera, Myrmeleontidae); they construct funnel-shaped pits in fine-grained soil or sand at rain-protected sites and ambush prey that fall into the trap (Wheeler 1930; Woodley and Swart 2017; Samocha and Scharf 2020). Vermileonidae is the only known dipteran family whose larvae capture prey by constructing pitfall traps in loose substrates (Wheeler 1930; Ludwig et al. 2001). Research on the family dates to the 19^th^ century (Macquart 1834) and initially focused on larvae, which comprise most of the life cycle, but studies of adult morphology developed mainly during the 20^th^ century (Majer 1988). Comprehensive morphological investigations of all life stages, including eggs, larvae, pupae, and adults, were not undertaken until the late 20^th^ century (Nagatomi et al. 1999; Ovtshinnikova 2014; Papp and Soltész 2019).

Molecular dating suggests a Middle Jurassic origin of Vermileonidae in India, followed by subsequent dispersal into the Palaearctic after the India–Laurasia collision in the Late Oligocene (Wiegmann et al. 2011; Wang et al. 2022). Fossil evidence indicates a mid-Cretaceous diversification and supports a two-stage evolutionary history: an early radiation in gymnosperm-dominated ecosystems, followed by angiosperm-driven diversification of derived lineages (Feng et al. 2025). Accordingly, genera with short mouthparts and fleshy labella (e.g. †*Crevermileo*, *Vermileo* Macquart, 1834, *Vermiophis* Yang, 1978, and *Vermitigris* Wheeler, 1930) are interpreted as basal, whereas nectar-feeding genera with elongated mouthparts diversified during the Late Mesozoic.

The genus *Vermitigris* previously comprised four described species. It was established on the basis of larval and pupal characters, with *V. fairchildi* Wheeler, 1930, from Sumatra, Indonesia, designated as the type species. Edwards (1932) transferred *Lampromyia orientalis* Brunetti, 1927 from Kuala Lumpur, Malaysia, to *Vermitigris* and provided the first detailed description of adult morphology for the genus. A specimen from southern India was tentatively identified as *V. orientalis* by Oldroyd (1947), but its identity remains unconfirmed (Bueno et al. 2021). Oldroyd (1947) described *V. infasciatus* from northern Borneo, Malaysia. Subsequently, *V. sinensis* Yang, 1988 was described from Guangxi, China, extending the confirmed distribution of the genus from southwestern Asia to mainland China.

In 1978, a series of wormlion larvae was collected from Yadong, southern Xizang, China, by the entomologist Fa-Sheng Li (Yang and Chen 1987). These larvae bear a longitudinal row of spines on the proleg, a character also observed in *V. fairchildi* larvae. However, in the absence of associated life stages, Yang and Chen (1987)—despite noting certain morphological differences (e.g. the number of spines on the proleg)—refrained from describing the material as a new species based solely on larval characters and emphasized the need for additional collections to resolve its identity. It is worth noting that the record from Yadong indicated in the distribution map by Nagatomi et al. (1999: fig. 325) was incorrectly attributed to *Vermiophis tibetensis* Yang & Chen, 1987, a misidentification later repeated by Li et al. (2024).

In 2025, an expedition to Yadong County, Xizang (Tibet), guided by earlier records, resulted in the collection of larvae, pupae, and adults of this species. Morphological examination and comparative analysis confirmed its placement in *Vermitigris*. The species differs markedly from the four previously known congeners in larval morphology, wing venation, and genital structures. Herein, *Vermitigris tsangyanggyatso* sp. nov. is described, with detailed illustrations of the larval, pupal, and adult stages, as well as notes on its biology. The phylogenetic and biogeographical implications for *Vermitigris* are also discussed.

## Materials and methods

The larvae were obtained and reared in fine-grained soil or sand taken from the collection site. Adults were obtained from a reared pupa or collected from wild habitats adjacent to larval colonies. All specimens are preserved in 75% ethanol and deposited in the Biological Science Museum, Dali University, Dali, China (BMDU). Dissected larval head skeleton and adult genitalia were macerated in 10% NaOH for 10 min, rinsed in tap water, and subsequently stored in glycerol within centrifuge tubes. Photographs were taken with a Nikon D850 digital camera in conjunction with a Nikkor AF-S Micro 105 mm f/2.8 lens (habitus), or a Canon R5 digital camera in conjunction with a Mitutoyo 10× M Plan Apochromatic Objective (for other images).

Observation and measurements were made under a Keyence VHX-7000 digital microscope. The distribution map was obtained from Maps-For-Free (https://maps-for-free.com) and modified with Adobe Illustrator CC. All resulting images were adjusted and combined using Adobe Photoshop 2022. Terminology follows that of Nagatomi et al. (1999) and Cumming and Wood (2017). Abdominal segments, terga, and sterna are abbreviated as A1, T1, and S1 (and so forth for other segments) in the text and figures.

## Results

### Taxonomy

**Order Diptera Linnaeus, 1758**

**Suborder Brachycera Zetterstedt, 1842**

**Family Vermileonidae Williston, 1886**

**Genus *Vermitigris* Wheeler, 1930**

#### Vermitigris tsangyanggyatso

Taxon classificationAnimaliaDipteraVermileonidae

Shan & Wang sp. nov.

6F437EBF-A2E8-5400-BC02-9B5734F6A9BE

https://zoobank.org/C8591EE3-54A0-48E7-B624-350963D2152B

**Figure 1.**
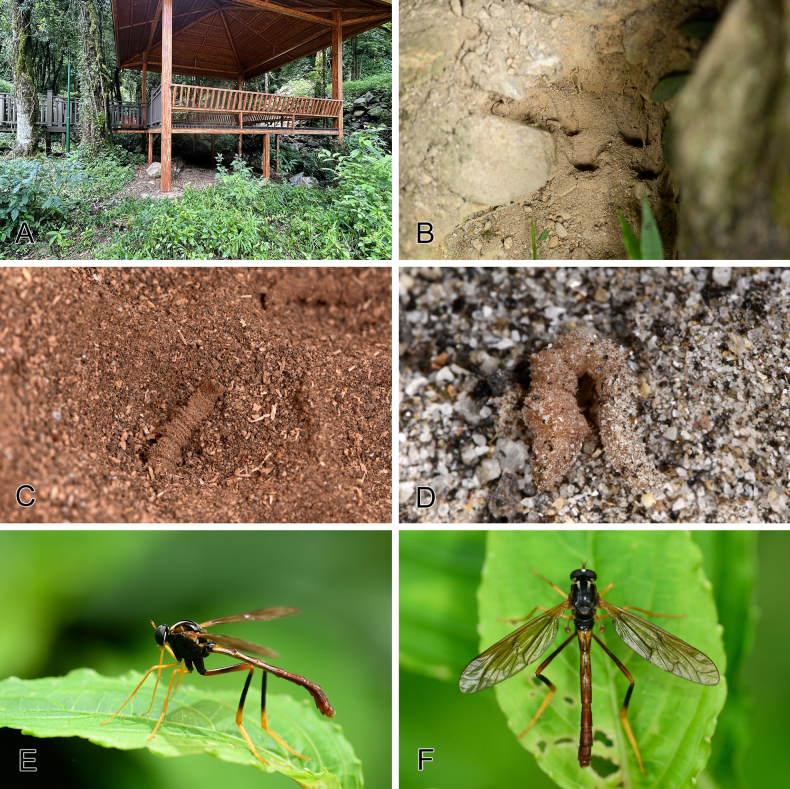
Habitats and habitus of *Vermitigris tsangyanggyatso* sp. nov. **A**. A pavilion shades a larval colony; **B**. Larval colony in fine-grained soil under a giant rock; **C**. Larva inhabiting wood debris produced by wood-boring insects under a pavilion; **D**. Larva inhabiting fine-grained river sands accumulated under a giant rock next to a river; **E, F**. Male resting on a plant leaf.

**Figure 2.**
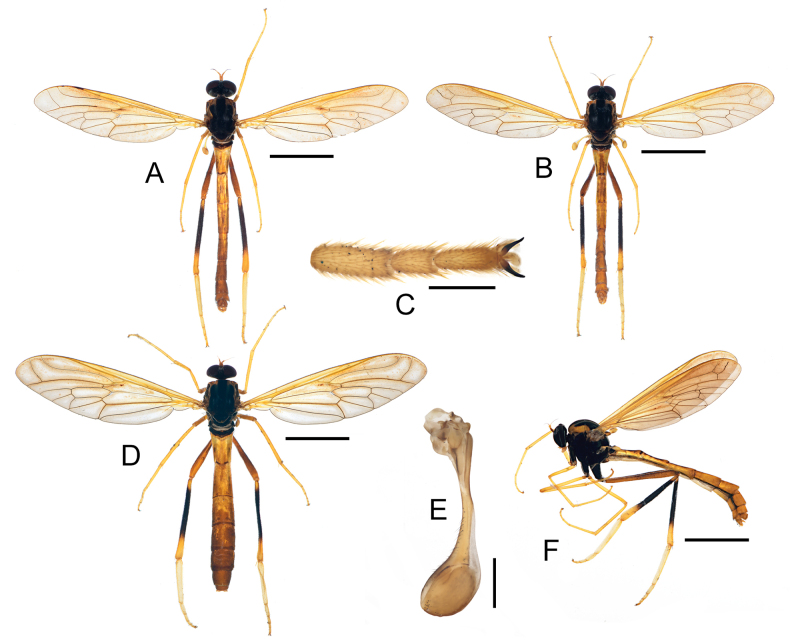
Morphological characters of *Vermitigris tsangyanggyatso* sp. nov. (I). **A**. Holotype, male, dorsal view; **B**. Paratype, male, dorsal view; **C**. Terminal portion of right hind leg of holotype, male, dorsal view; **D**. Paratype, female, dorsal view; **E**. Right halter of paratype, male, dorsal view; **F**. Paratype, male, left-lateral view. Scale bars: 2.0 mm (**A, B, D, F**); 0.2 mm (**C, E**).

**Figure 3.**
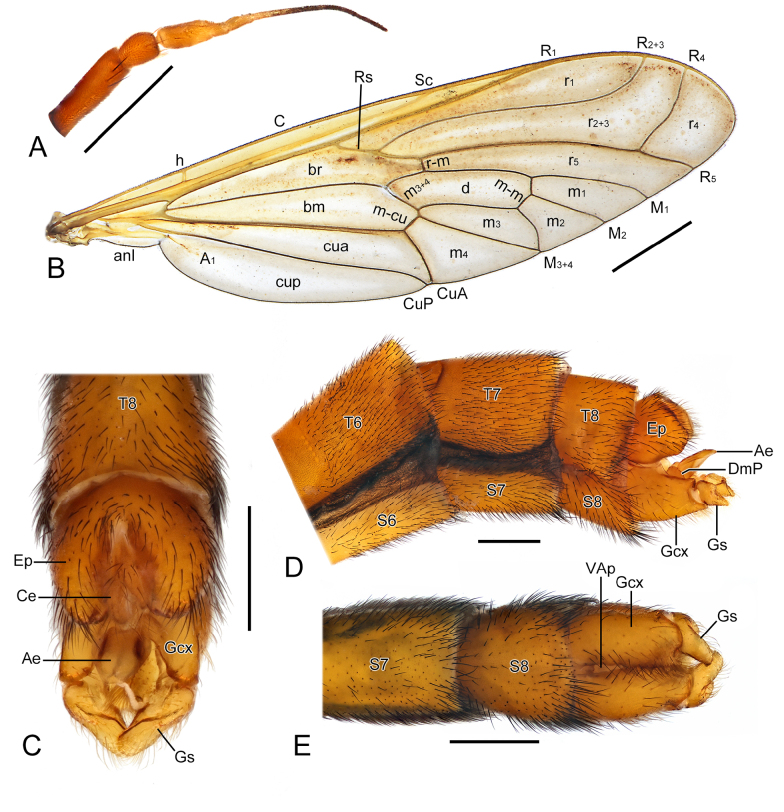
Morphological characters of *Vermitigris tsangyanggyatso* sp. nov. (II). **A**. Right antenna of paratype, male, dorsal view; **B**. Right wing of holotype, male, dorsal view; **C–E**. Terminal abdomen of paratype, male, dorsal, left-lateral, and ventral views. Abbreviations: Ae, aedeagus; Ce, cercus; DmP, dorsomedial projection; Ep, epandrium (T9); Gcx, gonocoxite; Gs, gonostylus; VAp, ventral aperture. Scale bars: 0.5 mm (**A, C–E**); 2.0 mm (**B**).

**Figure 4.**
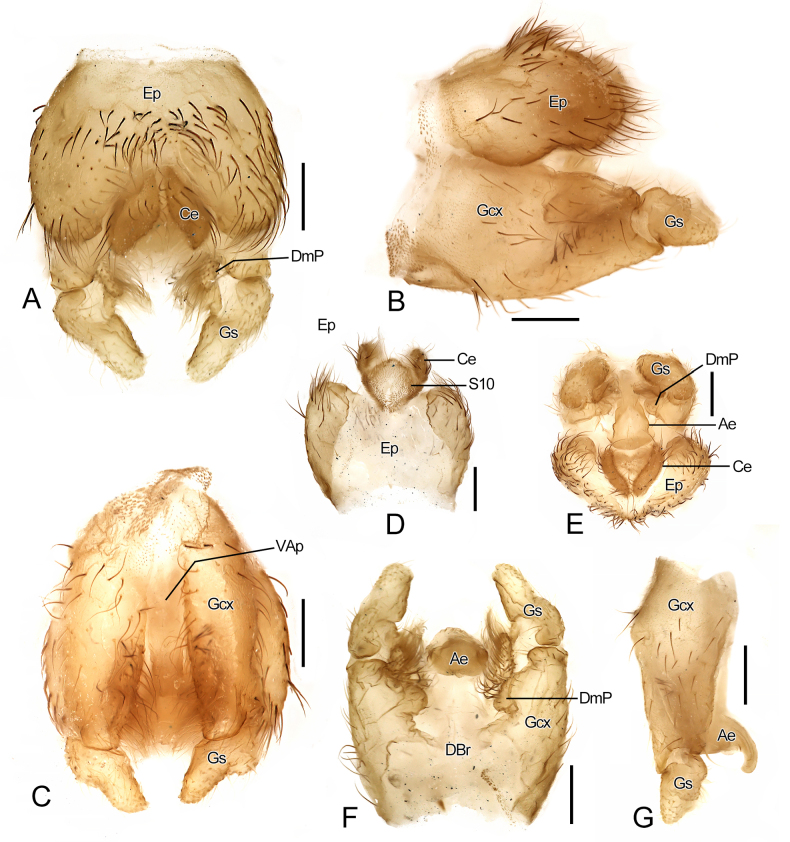
Morphological characters of *Vermitigris tsangyanggyatso* sp. nov. (III). Paratype, male. **A–C, E**. Genitalia, dorsal, left-lateral, ventral, and caudal views; **D**. Epandrium and A10, ventral view; **F, G**. Genitalia with epandrium removed, dorsal and left-lateral views. Abbreviations: Ae, aedeagus; Ce, cercus; DBr, dorsal bridge; DmP, dorsomedial projection; Ep, epandrium (T9); Gcx, gonocoxite; Gs, gonostylus; VAp, ventral aperture. Scale bars: 0.2 mm (**A–G**).

**Figure 5.**
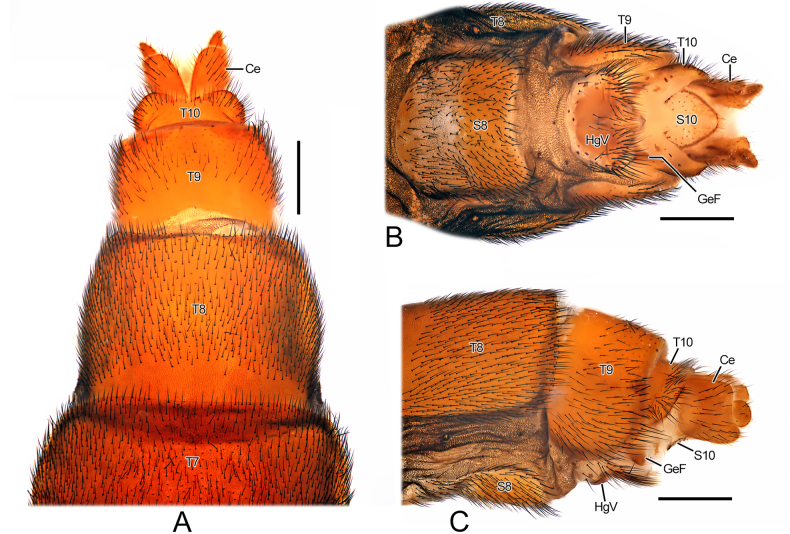
Morphological characters of *Vermitigris tsangyanggyatso* sp. nov. (IV). Paratype, female. **A–C**. Terminal abdomen, dorsal, ventral, and left-lateral views. Abbreviations: Ce, cercus; GeF, genital fork; HgV, hypogynial valve. Scale bars: 0.5 mm (**A–C**).

**Figure 6.**
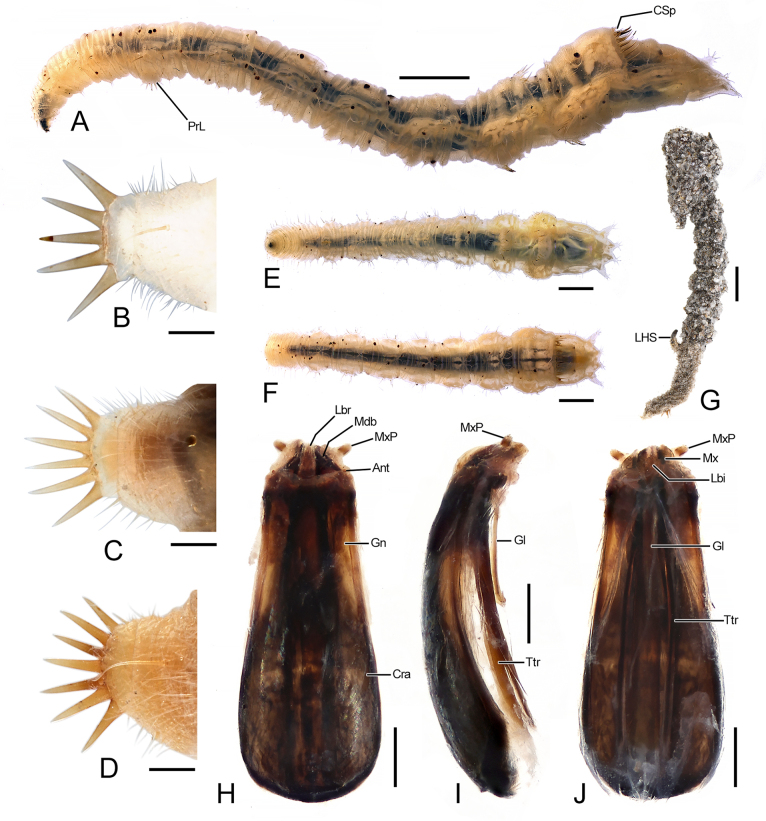
Morphological characters of *Vermitigris tsangyanggyatso* sp. nov. (V). Immature stages. **A, E, F**. Last instar larva, left-lateral, ventral, and dorsal views; **B–D**. Prolegs, left-lateral views; **G**. Pupa, right-lateral view; **H–J**. Larval head skeleton, dorsal, right-lateral, and ventral views. Abbreviations: Ant, antenna; Cra, cranium; CSp, comb-like spines; Gl, gula; Gn, gena; Lbr, labrum; LHS, larval head skeleton; Mdb, mandible; Mx, maxilla; MxP, maxillary palp; Ttr, tentorium. Scale bars: 2.0 mm (**A, E, F, G**); 0.2 mm (**B–D, H–J**).

**Figure 7.**
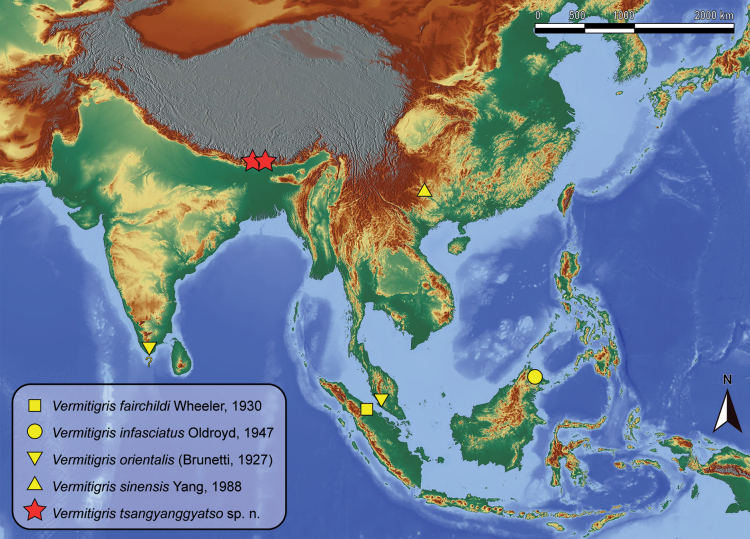
Distribution of *Vermitigris* spp. Question mark (?) indicates an unconfirmed record from India (Oldroyd 1947).

Figs 1, 2, 3, 4, 5, 6, 7

##### Chinese vernacular name.

仓央嘉措印穴虻.

##### Differential diagnosis.

In the larvae of this species, the marginal spines in the proleg are approximately as long as or only slightly shorter than the middle ones (Fig. 6B–D). However, in *V. fairchildi*, whose adults are unknown, the marginal spines are approximately half as long as the middle ones (Wheeler 1930: fig. 25j).

From *V. infasciatus* it can be differentiated by: (1) large body size with wing length exceeding 14 mm (vs 9 mm); (2) antennae mostly yellowish brown (vs dark); (3) abdomen lacks distinct stripes (vs with light longitudinal stripes extending from T2 to T5); (4) hind femur mostly reddish brown, hind tibia mostly dark brown and reddish brown in basal 1/10 and yellowish brown in distal 3/10 (vs femur uniformly dark brown, tibia yellowish brown in basal half and black in distal half). From *V. orientalis* it can be differentiated by: (1) meso- and metapleura black (vs mesopleura black but metapleura yellow); (2) abdomen uniformly yellowish brown (vs with light and dark rings); (3) R_2+3_ slightly curved (vs considerably curved); and in larvae, (4) proleg bears 5–7 spines (vs 3). From *V. sinensis* it can be differentiated by the absence of light and dark rings in the abdomen (vs presence), and R_4_ meets R_5_ at a subacute angle (vs almost right angle).

##### Type material.

***Holotype***. • ♂ (CNVer25TYG001), China, Xizang Tibetan Autonomous Region, Yadong County, western bank of Yadong (Chumbi) River, 27°13'54.99"N, 89°0'59.90"E, 1650 m elev., 23–31.VII.2025, leg. Chun-Mei Liao & Ji-Shen Wang.

***Paratypes***. • 3♂4♀ (CNVer25TYG002–008), same data; • 2♀ (CNVer25TYG009, 010), same location except 22–24.VIII.2025, leg. Si-Gui Fu; • 1♂ (CNVer25TYG011), China, Xizang Tibetan Autonomous Region, Dinggyê County, Zhêntang Town, northern bank of Ganma Tsangpo, 27°52'0.74"N, 87°24'56.33"E, 2500 m elev., 02.VIII.2025, leg. Chun-Mei Liao & Ji-Shen Wang.

##### Additional material.

28 larvae and three pupae, same data as the holotype. One pupa emerged as a male paratype (CNVer25TYG002).

##### Etymology.

The species is dedicated to Tsangyang Gyatso (1683–1706), the 6^th^ Dalai Lama, a Tibetan poet-monk from Xizang, renowned for his romantic verse and unconventional life. Noun in apposition.

##### Measurements (mm).

Adults: male body length 18.0–19.0, wing length 14.5–15.3, width 4.0–4.2 (*n* = 5); female body length 18.2–19.5, wing length 15.0–15.8, width 4.1–4.5 (*n* = 6). Larvae: final instar larvae body length 20–28 (*n* = 28). Pupae: body length 15–16 (excluding larval exuviae, *n* = 3).

##### Description.

**Male. *Head*** (Figs 1E, 1F, 2A, 2B, 2F, 3A). Antennae with scape and pedicel yellowish brown; flagellum darkened in terminal flagellomeres (arista). Scape long and cylindrical, approximately three times as long as pedicel and bearing three stout setae near apex dorsally; pedicel suboval; flagellum faintly four-segmented, with basal flagellomere nearly cylindrical; second and third flagellomere greatly narrowed and fourth slender, approximately as long as scape and pedicel combined. Vertex black with numerous setae. Frons dark brown with silver pollinosity. Proboscis short and fleshy.

***Thorax*** (Figs 1E, 1F, 2A–C, 2F). Meso- and metanotum mostly shining black, greatly humped. Mesonotum with two yellowish-brown stripes ending before scutellum, and large yellowish-brown spot near wing base. Pleura entirely black. Fore- and midlegs almost yellow except black coxae; hindlegs with coxa black, trochanter and femur reddish brown, tibia mostly dark brown and reddish brown in basal 1/10 and yellowish brown in distal 3/10, tarsi yellowish brown. Acropod with a pair of black claws and distinct empodium and pulvilli.

***Wings*** (Figs 1E, 1F, 2A, 2B, 2E, 2F, 3B). Wing membrane subhyaline and tinged with yellow, veins yellowish brown. R_4_ meets R_5_ at a subacute angle, R_5_ approximately 4/7 as long as R_4_. Cell m3 variable: open in holotype and one paratype, and closed in three other paratypes. Anal lobe narrow. Halters strongly clubbed.

***Abdomen*** (Figs 1E, 1F, 2A, 2B, 2E, 2F, 3C–E, 4A, 4B, 4D, 4E). T1 short, dark brown. T2–T8 yellowish brown to reddish brown, without longitudinal stripes or pale rings. Epandrium (T9) subtrapezoidal with V-shaped terminal emargination, with lateral margins greatly curled ventrad. Cerci short and broad.

***Genitalia*** (Figs 3C–E, 4). Gonocoxites divided by deep ventral aperture and connected by narrow dorsal bridge. Dorsomedial projection of gonocoxites rounded, bearing finger-like, setose process at base, directed caudad. Gonostyli short and blunt apically. Aedeagus tongue-shaped and curled ventrad at apex. Apodemes of aedeagus and gonocoxites indistinct.

**Female**. Habitus (Fig. 2D) similar to males but with much wider abdomen. Cerci two-segmented, with basal segment slightly wider than T10 in lateral view; distal segment much smaller. S10 nearly diamond-shaped.

***Genitalia*** (Fig. 5). Hypogynial valve approximately 3/4 as wide as S8 and bearing long setae in distal half. Genital fork greatly sclerotised.

##### Immature stages.

No eggs obtained.

Final instar larvae (Fig. 6A–F, H–J). Abdomen eight-segmented, with A7 widest. A1 with single semicircular proleg (pseudopod) bearing a longitudinal row of 5–7 spines (5, *n* = 8; 6, *n* = 13; 7, *n* = 7). Posterior border of T7 with two dorsal, comb-like rows of spines: posterior row with 14–18 strong spines, anterior row with 30–40 much weaker spines, only 1/5–1/3 length of strong ones. Four flat, broadly finger-shaped lobes on A8 posteriorly.

Larval head skeleton (Fig. 6A, H–J) greatly sclerotised, mostly retracted within first thoracic segment, approximately 2.5 times as long as wide. Cranium broad, widest at basal 1/3. Genae less sclerotised, exhibiting light colouration. Antennae (or remnants) extremely short. Labrum narrow. Mandibles subtriangular. Maxillae short, with two-segmented palps. Gula slender, approximately 1/3 length of cranium. Tentorium slender, at least twice as long as gula.

Pupae (Fig. 6G). Covered with fine grains of sand. Larval exuviae including head skeleton attached posteriorly.

##### Distribution.

(Fig. 7) China: southern Xizang (Dinggyê and Yadong counties). Because the collection sites are only a few kilometres from the national border, this species may also occur in nearby Bhutan, Nepal, and northern India.

##### Biological notes.

The larvae were collected from three different types of rain-protected microhabitats: (1) fine-grained soil under giant rocks (Fig. 1B); (2) a mix of soil and dense accumulation of wood debris produced by wood-boring insects beneath a pavilion (Fig. 1A, C); (3) fine-grained river sands accumulated under giant rocks next to the river (Fig. 1D). Field observations suggest that these wormlions mostly prefer the third type, as the first two usually harbor fewer individuals. When disturbed, the larvae exhibit a typical Ω-shaped defensive posture, as in other wormlions.

Adults were found resting on leaf surfaces around larval colonies on sunny days in Yadong (Fig. 1A, B). One male paratype from Dinggyê County, however, was collected around agricultural fields, with no larval colonies found nearby. This may suggest that this species has strong flight and dispersal abilities.

With a significantly shortened rostrum, *V. tsangyanggyatso* sp. nov. is similar to members of *Vermileo* and *Vermiophis*, which have no records of flower-visiting behaviour and are believed not to feed during the adult stage (Wheeler 1930; Nagatomi et al. 1999; Li et al. 2024).

### Key to species of *Vermitigris*

(Excluding *V. fairchildi* because the adults are unknown; modified from Nagatomi et al. 1999.)

**Table:** 

1	Abdomen with distinct light and dark rings	**2**
–	Abdomen lacking such rings	**3**
2	Antennal arista yellowish white; mesopleura black, metapleura yellow; T2–T6 ochraceous yellow in basal half and black in distal half	***V. orientalis* (Brunetti, 1927)**
–	Antennal arista dark brown to black; meso- and metapleura black; T2–T6 whitish grey in basal portion and black	***V. sinensis* Yang, 1988**
3	Antennae dark; abdomen with light longitudinal stripes extending from T2 to T5; hind femur uniformly dark brown, hind tibia yellowish brown in basal half and black in distal half	***V. infasciatus***
–	Antennae mostly yellowish brown; abdomen lacking such stripes; hind femur mostly reddish brown, hind tibia mostly dark brown and reddish brown in basal 1/10 and yellowish brown in distal 3/10	***V. tsangyanggyatso* sp. nov**.

## Discussion

To date, the members of this genus remain poorly documented. For instance, *Vermitigris fairchildi* is known only from the larvae and pupae (Wheeler 1930); *V. infasciatus* is known only from the male adults, with genitalia information lacking (Oldroyd 1947); *V. orientalis* is known from the male adults and larvae, but genitalia information is lacking (Brunetti 1927); and *V. sinensis* is known only from the females, larvae, and pupae (Yang 1988).

The pupae of *V. tsangyanggyatso* sp. nov. (Fig. 6G) are covered with fine grains of sand, different from those of *V. fairchildi*, which lack adherence of sand grains (Wheeler 1930: fig. 47). Yang (1988) also reported sand-grain adherence on the pupal cuticle of *V. sinensis*. Therefore, this condition likely results from differences in substrate conditions during larval ecdysis and is unlikely to be of diagnostic value. Due to the adherence of sand grains, we were unable to observe the distance between the antennal bases, which was considered a diagnostic character in *V. fairchildi* or even in the genus (Wheeler 1930; Yang 1988).

Based on morphological similarities, *V. tsangyanggyatso* sp. nov. is more similar to *V. orientalis* than to *V. infasciatus* and *V. sinensis*, owing to the absence of light and dark rings on the abdomen. This pattern suggests that southern Xizang, or more specifically the southern slope of the Himalayas, may serve as both a transitional zone and a biodiversity corridor facilitating the penetration of Oriental elements into Eurasia. According to the known distributional range (Fig. 7), many regions, such as northern India and the Indochina Peninsula, remain unsampled and may yield additional members of *Vermitigris*.

Wang et al. (2022) stated that wet forested regions have been shown to act as barriers to the dispersal of wormlion flies, corresponding to their absence from Central Africa, South America, and eastern North America. However, in the case of *Vermitigris*, most known species have been recorded from wet forested regions (except *V. sinensis*, which occurs in karst landscapes). This pattern may be explained by the presence of rain-protected microhabitats within these forests (for example, the space beneath a pavilion in Fig. 1A), which may provide a suitable microenvironment for the wormlions to construct their pitfall traps.

Four iNaturalist records of vermileonids can possibly be attributed to the genus *Vermitigris*: (1) a male from Booni, Chitral, Khyber Pakhtunkhwa, Pakistan (https://www.inaturalist.org/observations/230454888). This male exhibits antennae with a slender arista and a humped thorax, similar to *V. tsangyanggyatso* sp. nov. However, the yellowish pleura and coxae, black abdominal rings, and hind tibia yellow in the basal 1/3 and black in the distal 2/3 all suggest that this specimen represents an undescribed species of *Vermitigris*; (2) a female from Deramakot Forest Reserve, Telupid, Beluran, Sabah, Malaysia (https://www.inaturalist.org/observations/301754695). This individual matches the original description of *V. infasciatus*, including dark antennae, black pleura and hind femora, and a yellowish-brown abdomen lacking light rings. Its location is close to the type locality, Sandakan (Oldroyd 1947). Two additional nearby records (https://www.inaturalist.org/observations/210050346, https://www.inaturalist.org/observations/265264596) show larvae, which can probably be attributed to *V. infasciatus*.

Three other records from central and southern India (https://www.inaturalist.org/observations/267413682, https://www.inaturalist.org/observations/246137946, https://www.inaturalist.org/observations/246137947) show only pitfall traps, without accompanying images of the larvae or adults. Consequently, there is currently insufficient evidence to determine whether these observations represent wormlions (Vermileonidae) or antlions (Myrmeleontidae). One larval record from mainland Malaysia (https://www.inaturalist.org/observations/331252808) probably represents *V. orientalis*, although no definitive evidence is available.

## Conclusion

In this paper, we provide detailed illustrations and descriptions of the larval, pupal, and adult stages of *V. tsangyanggyatso* sp. nov., thereby expanding current knowledge of the morphology and biology of the genus *Vermitigris* and increasing the number of known species in this small genus from four to five, and all the extant species of the family from 66 to 67.

## Supplementary Material

XML Treatment for Vermitigris tsangyanggyatso

## Additional information

### Artificial Intelligence (AI) Use

Regarding the use of AI in the preparation of this manuscript, the authors declare the following: AI tools were used to check the grammar.

### Author ORCIDs

Li-Xia Shan https://orcid.org/0009-0002-5852-1733

Ji-Shen Wang https://orcid.org/0000-0002-0188-0228

### Data availability

All of the data that support the findings of this study are available in the main text.
